# Outcomes of COVID-19 Patients with Severe Hypoxemic Acute Respiratory Failure: Non-Invasive Ventilation vs. Straight Intubation—A Propensity Score-Matched Multicenter Cohort Study

**DOI:** 10.3390/jcm11206063

**Published:** 2022-10-14

**Authors:** Laura Pasin, Dario Gregori, Tommaso Pettenuzzo, Alessandro De Cassai, Annalisa Boscolo, Nicolò Sella, Giulia Lorenzoni, Federico Geraldini, Elisa Pistollato, Vito Marco Ranieri, Giovanni Landoni, Paolo Rosi, Paolo Navalesi, COVID-19 VENETO ICU Network

**Affiliations:** 1 Anesthesia and Intensive Care Unit, Padua University Hospital, 35128 Padua, Italy; 2 Unit of Biostatistics, Epidemiology and Public Health, Department of Cardiac, Thoracic, Vascular Sciences and Public Health, Padua University School of Medicine, 35128 Padua, Italy; 3 Department of Medicine (DIMED), Padua University School of Medicine, 35128 Padua, Italy; 4 Anesthesia and Intensive Care Medicine, Department of Medical and Surgical Science, Policlinico di Sant’Orsola, Alma Mater Studiorum-Università di Bologna, 40138 Bologna, Italy; 5 Department of Anesthesia and Intensive Care, IRCCS San Raffaele Scientific Institute, 20132 Milan, Italy; 6 Emergency Medical Services, Regional Department, AULSS 3, 30174 Venice, Italy

**Keywords:** SARS-CoV-2, non-invasive ventilation, mechanical ventilation, mortality

## Abstract

The best timing for endotracheal intubation in patients with coronavirus disease 2019 (COVID-19) hypoxemic acute respiratory failure (hARF) remains debated. Aim of this study is to compare the outcomes of COVID-19 patients with hARF receiving either a trial of non-invasive ventilation (NIV) or intubated with no prior attempt of NIV (“straight intubation”). All consecutive patients admitted to the 25 participating ICUs were included and divided in two groups: the “straight intubation” group and the “NIV” group. A propensity score matching was performed to correct for biases associated with the choice of the respiratory support. Primary outcome was in-hospital mortality. Secondary outcomes were length of mechanical ventilation, hospital stay and reintubation rate. A total of 704 COVID-19 patients were admitted to ICUs during the study period. After matching, 141 patients were included in each group. No clinically relevant difference at ICU admission was found between groups. In-hospital mortality was significantly lower in the NIV group (22.0% vs. 36.2%), with no significant difference in secondary endpoints. There was no significant mortality difference between patients who received straight intubation and those intubated after NIV failure. In COVID-19 patients with hARF it is worth and safe attempting a trial of NIV prior to intubation.

## 1. Introduction

The optimum timing for endotracheal intubation in adult patients with coronavirus disease 2019 (COVID-19) related severe hypoxemic acute respiratory failure (hARF) is debated [1].

Since the earliest stage of the COVID-19 pandemic, some guidelines suggested early intubation of critically ill patients with hARF consequent to Severe Acute Respiratory Syndrome Coronavirus 2 (SARS-CoV-2) infection [2,3], because of the risk of patient self-inflicted lung injury (p-SILI) and contagion of health care workers [4,5,6].

Nonetheless, firm evidence in favor of straight intubation in COVID-19 patients is still lacking since a recent meta-analysis including 9000 patients, Papoutsi et al. did not find significantly different rates of mortality between the patients who received early intubation and those intubated 24 h after intensive care unit (ICU) admission [7]. 

In this study, we aim to ascertain differences in mortality between patients who underwent a trial of non-invasive respiratory support [Continuous Positive Airway Pressure (CPAP) or Bilevel Positive Airway Pressure (BPAP)] and patients who received “straight” endotracheal intubation without a prior NIV attempt. Moreover, we assessed whether or not patients who failed NIV had a worse outcome, compared to patients who received straight intubation.

## 2. Materials and Methods

This multicenter, retrospective study was approved by the Institutional Ethical Committee of each of the 25 participating centers and the need for informed consent was waived. 

All consecutive adult patients with confirmed SARS-CoV-2 infection, admitted to ICU between February and April, 2020 were screened for eligibility. Exclusion criteria were: (1) patients with ceiling of treatment; (2) patients who underwent intubation before ICU admission; (3) incomplete data. Regional guidelines for respiratory management of COVID-19 patients were available for all ICUs of the network [8], but a clear-cut indication for the decision to intubate was not provided.

The following variables were prospectively collected and inserted in an online data acquisition system: demographic data (age, gender, body mass index (BMI), onset of symptoms); medical history (chronic disease, Charlson Comorbidity Index (CCI); Sequential Organ Failure Assessment (SOFA) score at ICU admission; respiratory parameters before endotracheal intubation, i.e., fraction of inspired oxygen (FiO_2_), arterial partial pressure of oxygen (PaO_2_), arterial partial pressure of carbon dioxide (PaCO_2_) and respiratory rate; presence of dyspnea; laboratory findings at ICU admission; days spent on invasive mechanical ventilation; reintubation; hospital lengths of stay; hospital mortality. Patients’ privacy was protected by assigning a de-identified patient code.

The primary outcome of our study was in-hospital mortality. Secondary outcomes were duration of invasive mechanical ventilation, reintubation rate and length of hospital stay.

We followed the Strengthening the Reporting of Observational Studies in Epidemiology (STROBE) statement guidelines for observational cohort studies [9] (Supplemental Table S3).

### Statistical Analysis

A propensity score matching was performed to correct for potential biases. Age, gender, CCI, BMI, the SOFA score at ICU admission and all variables resulting unbalanced between groups (*p* < 0.05 at univariate analysis) were matched. 

Normally distributed continuous data are reported as mean ± standard deviation (SD), while non-normal distributed data as median and interquartile range (IQR) and categorical variables as numbers and percentages. Comparisons between groups were performed with the Student *t*-test or the Mann–Whitney U test, for the continuous variables normally or non-normally distributed, respectively. Categorical data were compared using the Chi-square (or Fisher) test, as appropriate.

Subgroup analysis was performed to compare the outcomes of patients who received “straight intubation” and patients who failed NIV and required endotracheal intubation (“delayed intubation”).

Curves of cumulative incidence of in-hospital mortality were drawn to describe the trend of in-hospital mortality for both groups. The Gray’s test was used to assess differences between cumulative incidence functions. The observation period started at the day of hospital admission.

All statistical tests were 2-tailed and statistical significance was defined as *p* < 0.05. Statistical analysis was conducted using R (version 4.0.5).

## 3. Results

**Figure 1:**
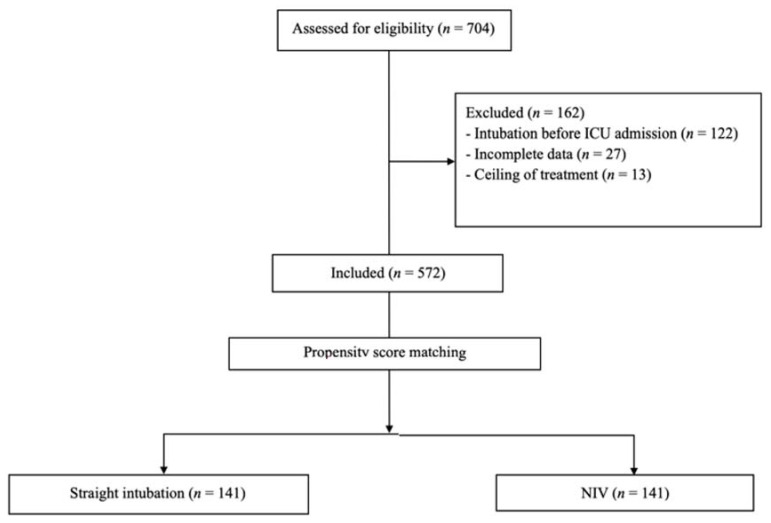
Flow diagram of screened and enrolled patients.

A total of 704 patients with confirmed SARS-CoV-2 infection were admitted during the study period. Among them, 162 were excluded and 542 were considered eligible. (Figure 1) Three-hundred and thirteen (57.8%) underwent intubation without previous NIV trial, and 229 (42.2%) received NIV prior to intubation for median 2 [1,2,3,4,5] days (Figure 1).

Baseline characteristics of the overall population and difference between groups at ICU admission are presented in the Supplemental Material (Supplemental Tables S1 and S2). The only significant difference between groups was a slightly greater SOFA score at ICU admission (5.00 [4.00–8.00] vs. 4.00 [3.00–6.00]) in the “straight” intubation group, and NIV group, respectively.

After propensity score matching, 141 patients were included in the “straight” intubation group and 141 patients in the NIV group. (Figure 1) Beyond weighted variables, no significant differences in demographic, clinical characteristics and respiratory parameters at ICU admission between groups were observed, except for a greater PaCO_2_ in the “straight intubation” group, compared to the NIV group (38.00 [34.00–45.00] vs. 36.00 [32.00–41.00] mmHg, respectively; *p* = 0.04) (Table 1).

**Figure 2:**
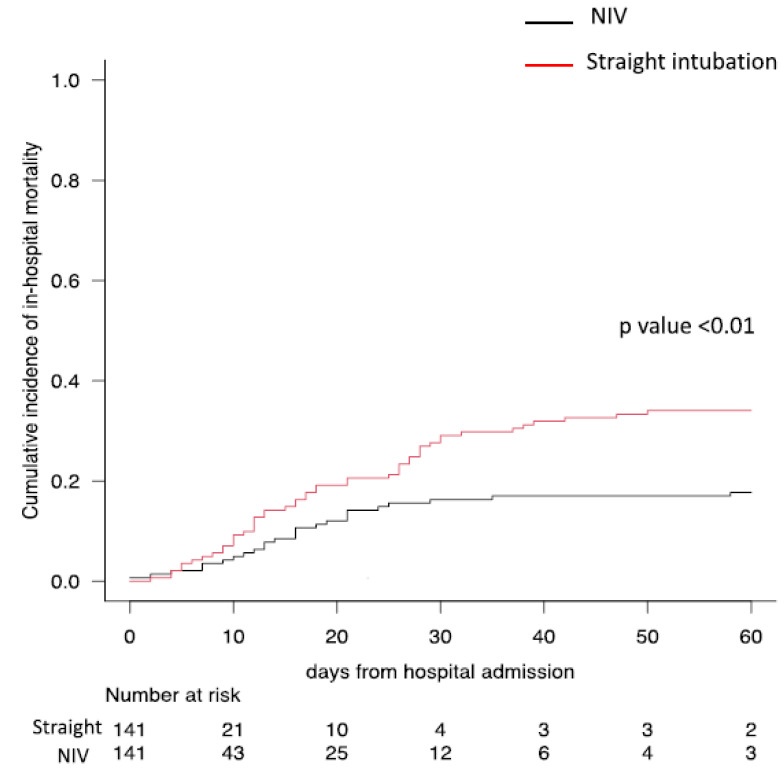
Difference in cumulative incidence of in-hospital mortality between straight intubation and NIV patients.

In-hospital mortality was significantly greater in the “straight” intubation group than in the NIV group (36.2% vs. 22.0%, respectively; *p* = 0.01), while no significant difference was observed with respect to length of invasive mechanical ventilation, reintubation rate and hospital length of stay (Table 2, Figure 2). 

**Figure 3:**
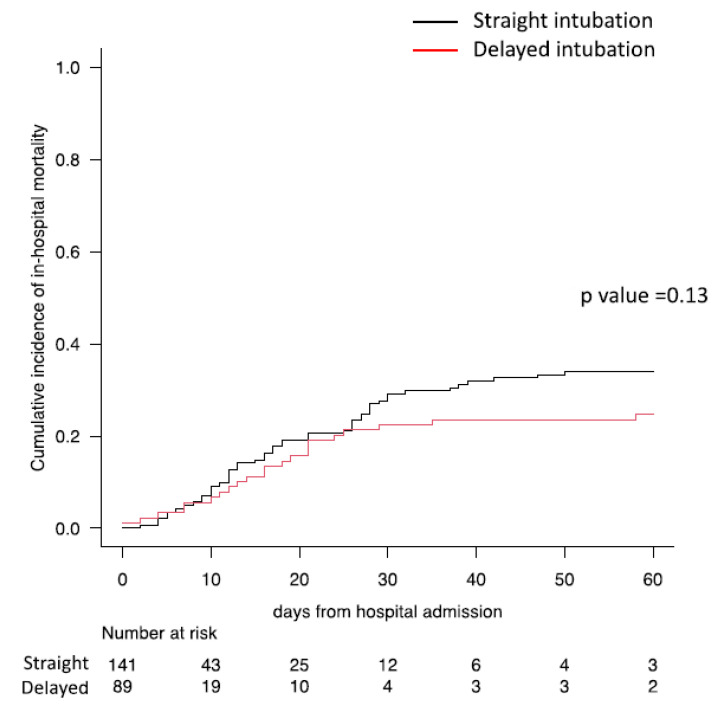
Difference in cumulative incidence of in-hospital mortality between straight and delayed intubation.

When comparing patients who received “straight” intubation and those who received delayed intubation after failing NIV, no difference in any clinical outcome was observed (Table 3, Figure 3).

## 4. Discussion

Our study shows that, regardless of baseline clinical conditions and potential medical confounders, attempting a NIV trial prior to intubation was associated to better survival of COVID-19 patients admitted to ICU for hARF, as compared to “straight intubation”. In addition, when comparing patients who received “straight intubation” and patients who failed NIV and required delayed intubation, we found no difference in any major clinical outcomes.

The timing of intubation in COVID-19 patients remains the subject of intense debate [1,10,11,12,13,14,15,16,17]. 

Papoutsi et al., in a recent systematic review and meta-analysis, did not find significantly different rates of mortality between the patients who received early intubation and those intubated 24 h after intensive care unit (ICU) admission. (45.4% vs. 39.1%, for early and late intubation, respectively). This trend was confirmed even considering an alternate definition of early/late intubation using as criterion a prior trial of HFNC or NIV. The authors concluded that early intubation did not reduce mortality and morbidity of COVID-19 patients with ARF, thus justifying a wait-and-see approach with non-invasive respiratory support before endotracheal intubation [7]. Noteworthy, the authors found no difference in mortality between patients who received intubation with or without a previous trial with non-invasive respiratory supports [7].

In keeping with previous studies, our results indicate that a wait-and-see approach could be the best option for the management of COVID-19 patients with hARF, at least in the ICU setting [11,16].

Three single center, prospective observational studies showed that late intubation was associated with increased ICU mortality [17,18,19]. However, in these investigations, the definition of ‘late intubation’ was based on the time between hospital admission or first respiratory support and endotracheal intubation (i.e., > or <24 or 48 h from hospital admission and > or <48 h from first respiratory support), irrespective of prior NIV use and location of application [17,18,19]. Indeed, previous work showed that the duration of NIV application in the ward before ICU admission was an independent risk factor of in-hospital mortality of COVID-19 patients with ARF [11,16].

Noteworthy, in the study by Vera et al. ‘late intubation’ was a risk factor for ICU mortality, as well as older age and severe hypoxemia, both adjusted by our propensity score matching model [18].

Our study has limitations. First, it is a retrospective study and therefore may be affected by a selection bias. Nonetheless, the influence of major confounding factors was limited by the use of the propensity score matching. Second, the outcomes we observed in our population do not probably reflect those of patients treated outside a pandemic condition and the use of non-invasive respiratory supports may not be representative of clinical practice in non-pandemic circumstances. Third, although guidelines for respiratory management of COVID patients were provided to all participating centres, the decision to intubate was left to the attending intensivists. In addition, the number of patients compared might be too small and may have hampered to see a significant difference. Moreover, data on clinical signs of respiratory distress (accessory respiratory muscles recruitment, diaphoresis, tachycardia, hypertension), of hemodynamic instability, lactate, etc. and their potential difference between the two groups were not available. We acknowledge that these data would have been useful to better understand what led physicians to directly intubate patients with apparently similar clinical characteristics. In fact, all these factors not only affect the decision to intubate but also indicate more severely ill patients that had a higher expected mortality. In this regard in fact, we found that among patients who were eventually intubated there were no differences in outcome. This probably indicates that some patients in the straight intubation group would manage to recover without intubation and this remains the great question in the literature: when to and when not to intubate. Unfortunately, when we compared the patients who were eventually intubated, the numbers were small to conclude on outcome differences.

## 5. Conclusions

Our findings indicate that in COVID-19 patients with hARF a trial of NIV in ICU might be of potential benefit and does not add to the risk of worsening patient outcome in the case of failure. 

## Figures and Tables

**Table 1:** Differences between groups at ICU admission after propensity score matching.

	Straight Intubation *n* = 141	NIV *n* = 141	*p*-Value
**Demographic and clinical characteristics, median [IQR]**			
Age (years)	68 (58–75)	66 (57–73)	0.43
Gender (male)	105 (74.5)	101 (71.6)	0.69
BMI (kg/m^2^)	28 (25–31)	28 (25–31)	0.94
Charlson Comorbidity Index	3 (2–5)	3 (2–5)	1.00
SOFA score at ICU admission	4 (3–5)	4 (3–5)	1.00
Onset of symptoms (days)	9 (6–13)	8 (6–11)	0.28
Hospitalization before ICU admission (days)	3.00 (1.00–5.00)	2.00 (1.00–5.00)	0.78
**Respiratory parameters at ICU admission, median [IQR]**			
Respiratory rate (breaths/min)	23.00 (16.00–30.00)	22.00 (17.50–30.00)	0.87
pH	7.45 (7.40–7.49)	7.43 (7.37–7.47)	0.07
PaO_2_/FiO_2_	114.00 (74.5–195.83)	120.71 (83.8–181.17)	0.32
PaCO_2_ (mmHg)	38.00 (34.00–45.00)	36.00 (32.00–41.00)	0.04
Presence of dyspnea, *n* (%)	75 (64.7)	66 (68.0)	0.66
**Chronic diseases, *n* (%)**			
COPD	8 (5.7)	10 (7.1)	0.86
Previous myocardial infarction	15 (10.7)	12 (8.5)	0.62
Cognitive decline	8 (5.7)	8 (5.7)	0.99
Complicated diabetes	6 (4.2)	2 (1.4)	0.28
Peripheral vascular disease	5 (3.5)	10 (7.1)	0.44
Moderate to severe CKD	7 (5.0)	4 (2.8)	0.37

Data are presented as number (%) or median (interquartile range). Abbreviations: NIV: non-invasive ventilation; BMI: body mass index; SOFA: sequential organ failure assessment; ICU: intensive care unit; SpO2: peripheral oxygen saturation; PaO_2_/FiO_2_: ratio between partial pressure of arterial oxygen and fraction of inspired oxygen; PaCO2: partial pressure of carbon dioxide; COPD: chronic obstructive pulmonary disease; CKD: chronic kidney disease.

**Table 2:** Differences in major outcomes between groups.

	Straight Intubation *n* = 141	NIV *n* = 141	*p*-Value
**In-hospital deaths, *n* (%)**	51 (36.2)	31 (22.0)	**0.01**
**Length of hospital stay, days**	29 (15–41)	25 (16–36)	0.37
**Length of invasive mechanical ventilation, days**	9 (5–14)	10 (7–14)	0.42
**Reintubations, *n* (%)**	9 (6.6)	10 (7.2)	0.99

Data are presented as number (%) or median (interquartile range). Abbreviations: NIV: non-invasive ventilation.

**Table 3:** Differences in major outcomes between patients who underwent “straight” intubation and delayed intubation (after NIV failure).

	Straight Intubation *n* = 141	Delayed Intubation *n* = 89	*p*-Value
**In-hospital deaths, *n* (%)**	51 (36.2)	26 (29.2)	0.32
**Length of hospital stay, days**	29.00 (15.00–41.00)	28.00 (18.00–40.75)	0.79
**Length of invasive mechanical ventilation, days**	10.00 (7.00–14.00)	9.00 (5.00–16.00)	0.54
**Reintubations, *n* (%)**	9 (6.6)	10 (11.5)	0.22

Data are presented as number (%) or median (interquartile range). Abbreviations: NIV: non-invasive ventilation.

## Data Availability

Data supporting reported results are available on request.

## Supplementary Materials

The following supporting information can be downloaded at: https://www.mdpi.com/article/10.3390/jcm11206063/s1, Table S1: Characteristics of overall population. Table S2: Differences in baseline demographic and clinical characteristics of patients at ICU admission. Table S3: STROBE Statement—Checklist.
