# Supplementary material for: Outcomes of COVID-19 Patients with Severe Hypoxemic Acute Respiratory Failure: Non-Invasive Ventilation vs. Straight Intubation—A Propensity Score-Matched Multicenter Cohort Study
Source: J Clin Med. 2022 Oct 14;11(20):6063. doi: 10.3390/jcm11206063 (PMC9605173; doi:10.3390/jcm11206063)
Supplement: Supplementary file 1 [file jcm-11-06063-s001.zip › jcm-1912548-supplementary.pdf]

Supplemental Material

Outcomes of COVID-19 patients with severe hypoxemic acute respiratory failure: non-invasive ventilation vs. straight intubation. A propensity score-matched multicenter cohort study.

INDEX

**Supplemental Table 1: Characteristics of overall population**	**Pag 2-3**
**Supplemental Table 2. Differences in baseline demographic and clinical characteristics of patients at ICU admission**	**Pag 4-5**
**Supplemental Table 3. STROBE Statement—Checklist.**	**Pag 6**
**Members of COVID-19 VENETO ICU Network**	**Pag 10-12**

Supplemental Table 1: Characteristics of overall population

	**Overall population, n=572**
Demographic and clinical characteristics, median [IQR]	
**Age (years)**	68 [58 - 75]
**Gender (male)**	415 (76.6)
**BMI (kg/m^2)**	28 [25 - 31]
**Charlson Comorbidity Index**	4 [2-5]
**SOFA score at ICU admission**	5 [3-7]
**Onset of symptoms (days)**	9 [6 - 12]
Respiratory parameters at ICU admission, median [IQR]	
**Respiratory rate (breaths/min)**	22 [18-30]
**PaO_2/FiO_2**	113.92 [77.88- 175.67]
**Presence of dyspnea, n (%)**	258 (65.0)
Treatments before ICU admission, n (%)	
**Length of hospitalization before ICU admission (days), median [IQR]**	2[1-5]
Antibiotic therapy	176 (32.5)
Corticosteroids	178 (38.0)
***Chronic diseases,* n(%)**	
COPD	41 (7.6)
Previous myocardial infarction	56 (10.4)
Cognitive decline	21 (3.9)
Complicated diabetes	36 (6.3)
Peripheral vascular disease	45 (8.3)
Moderate to severe CKD	31 (5.8)
Long-term medical treatments, n(%)	
Ace inhibitors	116 (21.6)
Angiotensin II receptor antagonists	71 (13.2)

Statins	106 (19.7)
Anticoagulants	54 (9.4)
<u>Laboratory findings at ICU admission, median [IQR]</u>	
WBCs (×10^9/L)	9[6.00-12.00]
PCT (ng/ml)	0.30 [0.00- 1.00]
Ferritin (ng/ml)	1422.00 [626.00- 2106.00]
IL-6 (ug/L)	87.00 [46.00- 194.00]
Fibrinogen (g/L)	6.00 [5.00- 7.70]
PT (%)	75.00 [64.00- 93.00]

Data are presented as number (%) or median [interquartile range]

Abbreviations: BMI: body mass index; SOFA: sequential organ failure assessment; ICU: intensive care unit; PaO_2/FiO_2: ratio between partial pressure of arterial oxygen and fraction of inspired oxygen; *COPD: chronic obstructive pulmonary disease; CKD: chronic kidney disease; WBC: white blood cells; PCT: procalcitonin; IL: interleukin; PT: prothrombin time; ICU: intensive care unit.*

Supplemental Table 2. Differences in baseline demographic and clinical characteristics of patients at ICU admission

	**straight intubation** n=313 (58%)	**NIV** n=229(42%)	*p-value*
Demographic and clinical characteristics			
**Age (years)**	68 [58- 75]	68 [58- 74]	0.83
**Gender (male)**	244 (78.00)	171 (74.70)	0.41
**BMI (kg/m^2)**	28 [26- 31]	28 [25- 31]	0.17
**Charlson Comorbidity Index**	4 [2-5]	4 [3- 5]	0.13
**SOFA score at ICU admission**	5 [4- 8]	4 [3- 6]	**<0.001**
**Onset of symptoms (days)**	9 [6-13]	9 [6- 12]	0.30
Respiratory parameters at ICU admission			
**Respiratory rate (breaths/min)**	22.00 [16.00- 30.00]	24.00 [20.00-30.00]	0.16
**PaO_2/FiO_2**	103.33 [75.78- 180.50]	118.33 [81.43-171.67]	0.08
**Presence of dyspnea**	142 (66.4)	116 (63.4)	0.60
Treatments before ICU admission			
**Length of hospitalization before ICU admission (days)**	2 [1- 5]	2 [1- 5]	0.81
Antibiotic therapy	99 (31.7)	77 (33.6)	0.69
Corticosteroids	94 (36.6)	84 (39.6)	0.61
***Chronic diseases,* n(%)**			
COPD	22 (7.0)	19 (8.3)	0.71
Previous myocardial infarction	31 (10.0)	25 (10.9)	0.43
Cognitive decline	8 (2.6)	13 (5.7)	0.10
Complicated diabetes	16 (5.1)	20 (8.7)	0.13
Peripheral vascular disease	21 (6.8)	24 (10.5)	0.11
Moderate to severe CKD	17 (5.5)	14 (6.1)	0.31
***Long-term medical treatments* n(%)**			
Ace inhibitors	71 (22.9)	45 (19.8)	0.46
Angiotensin II receptor antagonists	39 (12.6)	32 (14.1)	0.70
Statins	63 (20.3)	43 (18.9)	0.86
Anticoagulants	23 (7.4)	31 (13.5)	0.90

Laboratory findings at ICU admission			
WBCs (×10^9/L)	9.00 [6.00- 12.00]	8.00 [6.00- 12.00]	0.30
PCT (ng/ml)	0.30 [0.00- 1.00]	0.20 [0.00- 1.00]	0.44
Ferritin (ng/ml)	1518.00 [705.00-2368.00]	1300.50 [540.75-1992.75]	0.28
IL-6 (ug/L)	111.00 [59.00-215.50]	80.00 [31.25- 163.75]	0.31
Fibrinogen (g/L)	6.09 [5.00- 7.97]	6.00 [4.80-7.00]	0.15
PT (%)	76.00 [64.00-103.00]	75.00 [65.75-93.00]	0.81

Data are presented as number (%) or median [interquartile range]

Abbreviations: BMI: body mass index; SOFA: sequential organ failure assessment; ICU: intensive care unit; PaO_2/FiO_2: ratio between partial pressure of arterial oxygen and fraction of inspired oxygen; *COPD: chronic obstructive pulmonary disease; CKD: chronic kidney disease; WBC: white blood cells; PCT: procalcitonin; IL: interleukin; PT: prothrombin time; ICU: intensive care unit.*

Supplemental Table 3. STROBE Statement—Checklist.

	Item No	Recommendation	Page No
**Title and abstract**	1	(*a*) Indicate the study's design with a commonly used term in the title or the abstract	1-4
		(*b*) Provide in the abstract an informative and balanced summary of what was done and what was found	1-4
**Introduction**			
Background/rationale	2	Explain the scientific background and rationale for the investigation being reported	5
Objectives	3	State specific objectives, including any prespecified hypotheses	6
**Methods**			
Study design	4	Present key elements of study design early in the paper	6
Setting	5	Describe the setting, locations, and relevant dates, including periods of recruitment, exposure, follow-up, and data collection	6
Participants	6	(*a*) Give the eligibility criteria, and the sources and methods of selection of participants. Describe methods of follow-up	6, Figure 1
		(*b*) For matched studies, give matching criteria and number of exposed and unexposed	-
Variables	7	Clearly define all outcomes, exposures, predictors, potential confounders, and effect modifiers. Give diagnostic criteria, if applicable	6,7
Data sources/ measurement	8*	For each variable of interest, give sources of data and details of methods of assessment (measurement). Describe comparability of assessment methods if there is more than one group	-

Bias	9	Describe any efforts to address potential sources of bias	-
Study size	10	Explain how the study size was arrived at	Figure 1
Quantitative variables	11	Explain how quantitative variables were handled in the analyses. If applicable, describe which groupings were chosen and why	7
Statistical methods	12	(*a*) Describe all statistical methods, including those used to control for confounding	8
		(*b*) Describe any methods used to examine subgroups and interactions	8
		(*c*) Explain how missing data were addressed	8
		(*d*) If applicable, explain how loss to follow-up was addressed	8
		(*e*) Describe any sensitivity analyses	-
**Results**			
Participants	13*	(a) Report numbers of individuals at each stage of study—eg numbers potentially eligible, examined for eligibility, confirmed eligible, included in the study, completing follow-up, and analyzed	9, Figure 1
		(b) Give reasons for non-participation at each stage	Figure 1
		(c) Consider use of a flow diagram	Figure 1
Descriptive data	14*	(a) Give characteristics of study participants (eg demographic, clinical, social) and information on exposures and potential confounders	9
		(b) Indicate number of participants with missing data for each variable of interest	-

		(c) Summarize follow-up time (eg, average and total amount)	9
Outcome data	15*	Report numbers of outcome events or summary measures over time	10
Main results	16	(a) Give unadjusted estimates and, if applicable, confounder-adjusted estimates and their precision (eg, 95% confidence interval). Make clear which confounders were adjusted for and why they were included<br><br>(b) Report category boundaries when continuous variables were categorized<br><br>(c) If relevant, consider translating estimates of relative risk into absolute risk for a meaningful time period	10
Other analyses	17	Report other analyses done—eg analyses of subgroups and interactions, and sensitivity analyses	-
**Discussion**			
Key results	18	Summarize key results with reference to study objectives	11, 12
Limitations	19	Discuss limitations of the study, taking into account sources of potential bias or imprecision.<br><br>Discuss both direction and magnitude of any potential bias	12

Interpretation	20	Give a cautious overall interpretation of results considering objectives, limitations, multiplicity of analyses, results from similar studies, and other relevant evidence	12
Generalisability	21	Discuss the generalizability (external validity) of the study results	12
**Other information**			
Funding	22	Give the source of funding and the role of the funders for the present study and, if applicable, for the original study on which the present article is based	14

***Members of COVID-19 VENETO ICU Network*:**

Silvia De Rosa[1], Nicola Cacco[2], Chiara Pretto[2], Martina Tocco[2], Enrico Tamburini[2], Marina Munari[2], Fabio Baratto[3], Fabio Toffoletto[4], Giulio Andreatta[2], Leonardo Gandolfi[2], Alessandra Gadaldi[2], Nicolò Brumana[2], Edoardo Forin[2], Christelle Correale[2], Elisa Pesenti[2], Davide Fregolent[2], Pier Francesco Pirelli[2], Davide Marchesin[2], Silvia Crociani[2], Matteo Perona[2], Nicola Franchetti[2], Michele Della Paolera[2], Caterina Simoni[2], Gianlorenzo Golino[2], Tatiana Falcioni[2], Alessandra Tresin[2], Chiara Schiavolin[2], Aldo Schiavi[2], Sonila Vathi[2], Daria Sartori[2], Alice Sorgato[2], Federico Linassi[2], Eugenio Serra[2], Demetrio Pittarello[2], Ivo Tiberio[2], Ottavia Bond[2], Elisa Michieletto[2], Luisa Muraro[2], Arianna Peralta[2], Paolo Persona[2], Enrico Petranzan[2], Francesco Zarantonello[2], Eleonora Piasentini[2], Lorenzo Bernardi[5], Roberto Pianon[5], Flavio Badii[6], Enrico Bosco[7], Moreno Agostini[8], Paride Trevisiol[9] Antonio Farnia[10], Mario Peta[10], Mauro Antonio Calò[11], Marco Meggiolaro[12], Francesco Lazzari[13], Ivan Martinello[13], Giorgio Fullin[13], Francesco Papaccio[13], Alfeo Bonato[14], Camilla Sgarabotto[14], Francesco Montacciani[3], Parnigotto Alessandra[3], Giuseppe Gagliardi[15], Gioconda Ferraro[15], Luigi Ongaro[16], Marco Baiocchi[17], Vinicio Danzi[1], Paolo Zanatta[18], Simonetta Marchiotto[19], Silvia Bassanini[19], Massimo Zamperini[20], Ivan Daroui[20], Walter Mosaner[21], Luigi Ongaro[22], Lorella Altafini[23], Ezio Sinigaglia[24], Alessandra da Ros[24]

[1] U.O.C Anestesia e Rianimazione, Ospedale di Vicenza, Vicenza (VI), AULSS 8 Berica

[2] Azienda Ospedale-Università di Padova, Padova

[3] U.O.C. Anestesia e Rianimazione, Ospedali Riuniti Padova Sud, Monselice (PD), AULSS 6 Euganea

[4] U.O.C. Anestesia e Rianimazione, Ospedali di San Donà di Piave e Jesolo, San Donà di Piave (VE), AULSS 4 Veneto Orientale U.O.C.

[5] U.O.C. Anestesia e Rianimazione, Presidio Ospedaliero "San Martino", Belluno (BL), AULSS 1 Dolomiti

[6] U.O.C. Anestesia e Rianimazione, Ospedale di Vittorio Veneto, Vittorio Veneto (TV), AULSS 2 Marca Trevigian

[7] U.O.C. Anestesia e Rianimazione, Ospedale di Conegliano, Conegliano (TV), AULSS 2 Marca Trevigiana

[8] U.O.C. Anestesia e Rianimazione, Ospedale di Montebelluna, Montebelluna (TV), AULSS 2 Marca Trevigiana

[9] U.O.C. Anestesia e Rianimazione, Ospedale di Oderzo, Oderzo (TV), AULSS 2 Marca Trevigiana

[10] U.O.C. Anestesia e Rianimazione, Ospedale Ca' Foncello, Treviso, AULSS 2 Marca Trevigiana

[11] U.O.C. Anestesia, Rianimazione e Terapia Antalgica, Presidio Ospedaliero di Mirano, Mirano (VE) AULSS 3 Serenissima

[12] U.O.C. Anestesia e Rianimazione, Ospedale SS. Giovanni e Paolo, Venezia, AULSS 3 Serenissima

[13] U.O.C. Anestesia e Rianimazione, Ospedale dell'Angelo, Mestre (VE), AULSS 3 Serenissima

[14] U.O.C. Anestesia e Rianimazione, Ospedale di Cittadella, Cittadella (PD), AULSS 6 Euganea

[15] U.O.C. Anestesia e Rianimazione, Ospedali di Rovigo e Trecenta, Rovigo, AULSS 5 Polesana

[16] U.O.C. Anestesia e Rianimazione, Ospedale Alto Vicentino, Santorso (VI), AULSS 7 Pedemontana

[17] U.O.C. Anestesia e Rianimazione, Ospedale San Bassiano, Bassano del Grappa (VI), AULSS 7 Pedemontana

[18] U.O. Anestesia e Rianimazione A, Azienda Ospedaliera Universitaria Integrata Verona, Verona

[19] U.O.C Anestesia e Rianimazione, Ospedale Magalini di Villafranca, Legnago (VR), AULSS 9 Scaligera

[20] Dipartimento di Anestesia, Rianimazione e Terapia Antalgica, IRCCS Sacro Cuore-Don Calabria, Negrar

[21] U.O. Terapia Intensiva, Ospedale P. Pederzoli – Casa di Cura Privata SpA, Peschiera sul Garda (VR)

[22] U.O.C. Anestesia e Rianimazione, Ospedale Alto Vicentino, Santorso (VI), AULSS 7 Pedemontana

[23] U.O.C. Anestesia, Rianimazione e Terapia Antalgica, Presidio Ospedaliero di Dolo, Dolo (VE), AULSS 3 Serenissima

[24] Anestesia e Rianimazione, Ospedale Mater Salutis di Legnago, Legnago (VR), AULSS 9 Scaligera
